# Electroanalytical Techniques for the Detection of Selenium as a Biologically and Environmentally Significant Analyte—A Short Review

**DOI:** 10.3390/molecules26061768

**Published:** 2021-03-22

**Authors:** Miroslav Rievaj, Eva Culková, Damiána Šandorová, Zuzana Lukáčová-Chomisteková, Renata Bellová, Jaroslav Durdiak, Peter Tomčík

**Affiliations:** Electroanalytical Chemistry Laboratory, Department of Chemistry and Physics, Faculty of Education, Catholic University in Ružomberok, Hrabovská cesta 1, SK-034 01 Ružomberok, Slovakia; miroslav.rievaj@ku.sk (M.R.); damiana.sandorova941@edu.ku.sk (D.Š.); zchomistekova@gmail.com (Z.L.-C.); renata.bellova@ku.sk (R.B.); jaroslav.durdiak@ku.sk (J.D.); peter.tomcik@ku.sk (P.T.)

**Keywords:** selenium, stripping voltammetry, environment, electroanalysis, deposition

## Abstract

This short review deals with the properties and significance of the determination of selenium, which is in trace amounts an essential element for animals and humans, but toxic at high concentrations. It may cause oxidative stress in cells, which leads to the chronic disease called selenosis. Several analytical techniques have been developed for its detection, but electroanalytical methods are advantageous due to simple sample preparation, speed of analysis and high sensitivity of measurements, especially in the case of stripping voltammetry very low detection limits even in picomoles per liter can be reached. A variety of working electrodes based on mercury, carbon, silver, platinum and gold materials were applied to the analysis of selenium in various samples. Only selenium in oxidation state + IV is electroactive therefore the most of voltammetric determinations are devoted to it. However, it is possible to detect also other forms of selenium by indirect electrochemistry approach.

## 1. Introduction

Selenium occurs naturally at low concentrations in the Earth’s crust, water and atmosphere. It is released into the atmosphere from various natural and anthropogenic sources: by soil weathering, coal, oil, wood and biomass burning, smelting of non-ferrous metals as well as from the agricultural products production and their usage [1]. Selenium and its compounds are used in industry as additives in metal alloys, pigments, glass decolorizers, photoreceptors in copying machines, semiconductors, and also in photoelectrochemical cells and photocells [2]. The natural occurrence of selenium in nature can vary considerably, that there are areas with high concentrations of selenium on the one hand and very low concentrations on the other. This fact also has an effect on what doses of selenium are beneficial in the given area—both for plants, animals and humans. The highest permitted content of Se in drinking water is 10 µg L^−1^ [3]. Selenium is a substantial element for the normal growth of animals and has a daily recommended level of less than 1 mg for humans [4].

Selenium is a part of selenoproteins formed in biological systems by the incorporation of modified amino acids like selenocysteine or selenomethionine. It protects cells from oxidative damage and viral infections, influences the metabolism of thyroid hormones and has anticarcinogenic activity [5]. Selenium deficiency in the human body may lead to cirrhosis, carcinoma, Keshan and Kashin-beck disease, but at increased intake levels (above 3 mg/day) it has toxic effects and causes oxidative stress, resulting in the chronic disease called selenosis. The toxicity of selenium is caused by its reactivity with thiols, thus affecting the function of DNA repair proteins leading to the formation of DNA-damaging free radicals. Long-term consumption results in structural defects, brittle hair, stratified nails and pulmonary edema [6].

In the environment, selenium may occur in different oxidation states: selenide Se(−II), elemental selenium Se(0), selenite Se(IV) and selenate Se(VI). In natural waters, it is present dominantly in the Se(IV) and (VI) oxidation states, but may also be in a few organic forms such as selenoaminoacids [7]. Selenium in oxidation state + IV is the most toxic and the one and only electroactive form of Se [8]. It is approximately twenty times more toxic than selenate due to its mobility, distribution, and bioavailability. The electrochemical determination of the various oxidation forms of selenium can be done only after their transformation to Se(IV) state by oxidation or reduction. This process must be done with care to avoid any loss of part of the selenium during the decomposition or treatment of the sample [9]. 

This article will provide an overview of the developments in the analysis of different forms of selenium in various samples by electroanalytical methods using voltammetric sensors based on different pure and modified materials.

## 2. Trace Analysis of Selenium on Hanging Mercury Drop and Thin Film Electrodes

Mercury is a very favored electrode material with which most electrochemists begin their studies because almost all electrode reactions are reversible on it, leading to regular nicely developed registered curves. Cathodic, adsorption and anodic stripping voltammetry are the three best known techniques for the determination of selenium with the highest sensitivity among all currently available analytical techniques. In the case of these methods, the signal increases radically by introducing an accumulation step in which elemental selenium is deposited on the electrode surface. When deposited selenium is dissolved back, it is important to reduce the noise current to achieve lower detection limits. In addition, it shortens the electrodeposition time and the total time consumed by whole analysis [10].

A hanging mercury drop electrode (HMDE) is mainly used for the direct determination of Se(IV) in cathodic stripping voltammetry (CSV) [11]. A suitable medium could be 0.1 mol L^−1^ HClO_4_, because in the presence of ClO_4_^−^ions the mercury electrode can be polarized with positive potentials without dissolving any electrode material. At negative potentials (−0.35 V) selenium is accumulated, while at positive potentials (+0.3 V) the surfactants are removed from the electrode. The calibration curve was found linear from 1 × 10^−9^ mol L^−1^ to 4 × 10^−8^ mol L^−1^ for an accumulation time of 180 s. The relatively low detection limit of 4 × 10^−10^ mol L^−1^ was obtained allowing such the detection of selenium (IV) in river water [12].

Grabarczyk et al. have described a simple technique for the trace analysis of Se(IV) in natural lake waters containing a high concentration of surfactants and humic species using a CSV method. Selenium was first accumulated on HMDE from a sample in a supporting electrolyte containing 0.1 mol L^−1^ HClO_4_ and 4 × 10^−4^ mol L^−1^ Cu(NO_3_)_2_ at a potential of −0.35 V vs. SCE for 30 s as Cu_2_Se, which was subsequently dissolved by differential pulse cathodic scanning of the potential towards −0.7 V vs. SCE. The interferences of dissolved organic substances were suppressed with Amberlite XAD-7 resin added directly into the voltammetric cell. A linear signal was registered in a concentration range from 2 × 10^−9^ to 2 × 10^−7^ mol L^−1^ with a relative standard deviation (RSD) of 3.7%. The detection limit (LOD) was estimated to be 7.8 × 10^−10^ mol L^−1^ for an accumulation time of 30 s. The proposed method is suitable for the direct analysis of Se(IV) in natural water samples without matrix removal or any sample preparation [13].

The signal-to-noise ratio can be improved further by optimization of the experimental parameters influencing the square-wave shape in traditional square-wave voltammetry (SWV) due to background current minimization. This approach was used for the determination of selenium (IV) by cathodic stripping voltammetry on HMDE in a solution of 0.1 mol L^−1^ nitric acid containing Cu(II) ions. A quasi-reversible kinetic copper selenide reduction process was observed with a peak current dependence on the frequency of the square-wave. This modified square-wave cathodic stripping voltammetry (SWCSV) technique has a detection limit of 8 × 10^−12^ mol L^−1^ for an accumulation time of 5 min and a relatively wide linear dynamic concentration range of Se(IV) from 1 × 10^−11^ to 1 × 10^−6^ mol L^−1^. The metal ions Mo(VI), Pb(II), Ni(II), Zn(II), Cr(VI) present in analyzed solution at 1000-fold molar excess did not interfere with the Se(IV) signal. This kind of selenium detection at trace and ultratrace levels is applicable for the analysis of biological and environmental samples [14].

Ultrasensitive determination of selenium was also performed in the presence of rhodium ions by cathodic stripping voltammetry based on the accumulation and subsequent reduction of the Rh_2_Se_3_ layer on a hanging mercury drop electrode. The cathodic stripping peak of accumulated Rh_2_Se_3_ was very sharp and 10 to 50 times higher than in the case of Cu_2_Se or HgSe stripping. For the selenium content a very low detection limit of 6 × 10^−12^ mol L^−1^ was reached after 3 min of accumulation at −0.2 V in 0.1 mol L^−1^ sulfuric acid as supporting electrolyte containing 9.7 × 10^−8^ mol L^−1^ rhodium. This methodology was applied to the analysis of groundwater and river water [15] as examples of real samples.

Microwave digestion is attractive for the decomposition of complex biological samples due to its rate, the use of small amounts of reagents and practically no loss of analyte compared to conventional methods. Prasad et al. described the trace determination of selenium in biological tissues with Cu_2_Se formation on HMDE. Microwave digestion of bovine liver was performed with HNO_3_, followed by HClO_4_ addition prior to selenium detection by square-wave cathodic stripping voltammetry. The detection limit of 1.3 × 10^−11^ mol L^−1^ and the RSD of 1.8% for Se(IV) were determined [16].

In recent years, speciation analysis of selenium has received attention of electrochemists. For example, it can be performed using cathodic stripping voltammetry on HMDE. The method is based on electrodeposition of Se(IV) with Cu(II) ions and the determination of Se(VI) after UV irradiation of the sample required for the photolytic reduction of selenium (Se(VI) to Se(IV)) in 0.1 mol L^−1^ HCl. The LOD reached 3.8 × 10^−10^ mol L^−1^ during 240 s deposition reaching relative standard deviation of 6.19% for 6.3 × 10^−8^ mol L^−1^ solution of Se(IV). A linear calibration range was observed from 1.3 × 10^−8^ to 1.3 × 10^−6^ mol L^−1^. The photolytic reduction of Se(VI) to electroactive species Se(IV) had yields from the range of 91.7% to 112.9%. Seawater, hydrothermal and hemodialysis fluids served as real samples [17].

In water samples, Se(IV) and Se(VI) were determined by cathodic stripping voltammetry of copper selenide also on a hanging mercury drop electrode but using an automated flow system. This system allows high sample throughput and minimal sample pretreatment. Replacement of the electrolyte used reduces the matrix effect during the voltammetric determination. One hour UV irradiation of the sample in alkaline medium (pH 11.0) was performed before reduction of Se(VI) to Se(IV). The Se(VI) content was determined from the difference between the total Se and Se(IV) content determined previously. The linear range of measurements was from 1.3 × 10^−8^ to 1.9 × 10^−6^ mol L^−1^ with a detection limit of 4.4 × 10^−9^ mol L^−1^ and an RSD of 1.4% for 6.3 × 10^−8^ mol L^−1^ solution of Se(IV). UV irradiation served both for the reduction of Se(VI) and also for the decomposition of the organic species present in water samples. The combination of these two processes allowed the specialization of Se(IV) and Se(VI), as well as the determination of total selenium with relatively high content of organic interferences [18].

Voltammetric detection of organic compounds of selenium with close half-wave potentials-selenourea (Se-U) and selenocystamine (Se-CM) was performed after their separation by ion exchange. Se-CM remained on the Purolite C 100 H cation exchange resin, while selenourea passed into effluent and was determined by square wave cathodic stripping voltammetry in a basic (Na_2_CO_3_) electrolyte on HMDE. Se-CM was then eluted from the cation exchange resin with 4 mol L^−1^ HCl and SWCSV in HCl solution and was also analyzed. Low detection limits of 3.8 × 10^−9^ mol L^−1^ for Se-CM and 2.5 × 10^−8^ mol L^−1^ for Se-U were obtained without the influence of interfering compounds with a similar structure such as urea, S-urea and S-cystamine. A procedure for the separation and electrochemical detection of inorganic (Se(IV) and Se(VI)) and organoseleniumcompounds (Se-U, Se-CM, (CH_3_)_2_Se_2_ and (CH_3_)_2_Se) has also been developed and successfully applied to certified reference materials, environmental soil samples and urine samples. (CH_3_)_2_Se_2_ in the organic phase as well as Se(IV) in the aqueous phase were determined directly and Se(VI) and (CH_3_)_2_Se were determined indirectly after suitable leaching. Methylated forms of selenium (Me_2_Se, Me_2_Se_2_) are formed by microorganisms in an aqueous medium [19].

The thin film mercury electrode (TFME) represents a more environmentally acceptable variant due to its substantially lower consumption of mercury in comparison to classical HMDE. Differential pulse cathodic stripping voltammetric (DPCSV) determination of selenium (IV) was performed using TFME on glassy carbon in 0.1 mol L^−1^ HClO_4_ solution containing 0.02 mol L^−1^ thiocyanate ions. The presence of thiocyanate ions increased the peak height of the selenium and also shifted it to more positive potentials. SCN^−^ ions are adsorbed on the electrode surface and thus catalyze the electron transfer between copper selenide and electrode. The LOD value was determined to be 9.5 × 10^−10^ mol L^−1^ for Se(IV) with an RSD of 5.2%. The interference of Cd(II), As(III), Zn(II), Fe(III) and Pb(II) ions on the selenium signal was also studied. In the presence of lead ions, the selenium peak was decreased and a new signal appeared at a more negative potential (−0.75 V) than in the reduction of Cu_2_Se (−0.65 V vs. Ag / AgCl). This signal corresponds to PbSe reduction and can also be used for selenium (IV) determination. The influence of other ions was negligible [20].

Thin film mercury electrode modification with copper leads to an increase of reproducibility and sensitivity. Cu(II) was reduced and formed a mercury-copper film on a glassy carbon electrode (GCE) allowed the analysis of Se(IV) in a wide concentration range from 1 × 10^−9^ to 1 × 10^−6^ mol L^−1^ by square- wave cathodic stripping voltammetry in 0.1 mol L^−1^ HNO_3_. During the accumulation step, copper selenide was electrolytically deposited on the abovementioned film. A detection limit of 8 × 10^−10^ mol L^−1^ was determined for five minutes of accumulation [21].

Better resistance to interferences caused by surfactants is offered by a chemically modified TFME electrode with photooxidized 3,3’-diaminobenzidine (ODAB) and with the perfluorinated polymeric anion exchanger Tosflex. The mixture of Tosflex and ODAB was placed on a glassy carbon electrode, with a thin mercury film. The ODAB was electrochemically reduced in the mercury film while selenium (IV) was accumulated on the electrode surface reacting with the reduced ODAB to form a complex. A linear dynamic concentration range from 6.3 × 10^−9^ to 6.3 × 10^−7^ mol L^−1^ was observed for five minutes of deposition and the detection limit was estimated to be 1.3 × 10^−9^ mol L^−1^. Selenium content in seawater by the SWCSV technique was determined by this method [22].

## 3. Modern Sensing Platforms for the Detection of Selenium Based on Solid Electrode Materials

**Figure 1:**
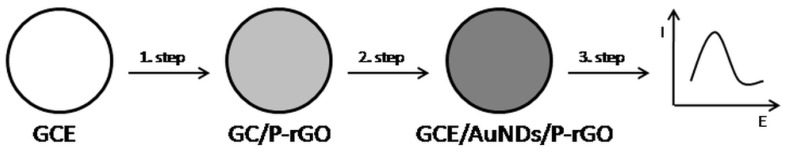
Schematic depiction of AuNDs/P-rGO-modified electrode preparation: 1. electrodeposition of reduced graphene oxide and Prussian blue (rGO@PB) films on GCE electrode using cyclic voltammetry with a potential range from −1.6 to +1.0 V; 2. electrodeposit Au nanodendrites at a constant potential of −0.2 V; 3. detecting Se(IV) using SWV in a potential range from −0.2 to +0.6 V.

Wei et al. presented in their work a mercury-free sensor for the analysis of Se(IV). It is based on a glassy carbon electrode modified with gold nanodendrites (AuNDs) and perforated reduced graphene oxide (P-rGO) (Figure 1). The composition of the modifier after electrochemical deposition was characterized by scanning electron microscopy. Gold nanomaterials are characterized by excellent conductivity and graphene is used because of its high thermal, large specific surface and unique catalytic properties. Square-wave anodic stripping voltammetry (SWASV) revealed a low LOD of 9 × 10^−10^ mol L^−1^ for Se(IV) and wide linear concentration region from 3 × 10^−9^ to 3 × 10^−7^ mol L^−1^. The proposed sensor was applied for the determination selenium in seawater as well as in standard samples of artificial seawater with different salinities [23].

On a glassy carbon electrode modified with Bi/Hg film at an open circuit spontaneous accumulation of Se(IV) was studied by differential pulse adsorption cathodic stripping voltammetry (DPAdsCSV) in 0.1 mol L^−1^ HCl. The Bi/Hg film electrode showed a strong adsorption capacity to Se(IV) during self-accumulation and increase its response and sensitivity during the cathodic stripping process. The limits of detection and quantification under optimal conditions were 8.9 × 10^−10^ mol L^−1^ resp. 3.2 × 10^−9^ mol L^−1^. This sensing platform is suitable to analyze selenium in vegetables, fruits and water samples providing reliable results verified by inductively coupled plasma optical emission spectrometry (ICP-OES) as reference technique [24].

Adsorption cathodic stripping voltammetry on a carbon paste electrode (CPE) may also be combined with a medium exchange in the case of Se(IV) determination. The method is based on the formation of a piazselenol complex from Se(IV) and 2,3-diamino- naphthalene at pH = 2 in a solution of 0.01 mol L^−1^ HCl and 0.1 mol L^−1^ KCl. After spontaneous non-electrochemical accumulation at the open circuit, the complex was stripped out after changing the medium to 0.1 mol L^−1^ HNO_3_ and 0.1 mol L^−1^ KNO_3_, into which the working electrode was transferred. The analyte was determined with accuracy better than 8% and a limit of quantification 1.25 × 10^−7^ mol L^−1^ was achieved after 20 min of deposition time. CPE associated with a medium exchange system reduces the interferences of different classes of surfactants in environmental samples and increases the selectivity in comparison to conventional cathodic stripping voltammetry on mercury electrodes [25].

Electrochemical trace determination of selenium can also be performed on a pencil graphite electrode (PGE) modified with a composite film made from acetophenone (2,4-dinitrophenyl)hydrazone (ADH) and polypyrrole (PPy) combined with copper nanoparticles (CuNPs) as electrocatalysts. A simple procedure based on cyclic voltammetry was used for simultaneous electropolymerization of PPy, incorporation of ADH and electrodeposition of CuNPs on the surface of PGE. The resulted modified electrode showed excellent stability, electrocatalytic activity with favorable electrochemical parameters for the reduction of Se(IV) to Se(0) in Britton-Robinson buffer (pH 2) at a potential of −0.85 V (vs. SCE). The electrochemical response for the above mentioned analyte was linear in the range of concentrations from 5 × 10^−8^ to 1.1 × 10^−7^ mol L^−1^ with a detection limit of 1.7 × 10^−8^ mol L^−1^ if a square wave variant of stripping voltammetry is used before subsequent application to samples of milk and waste water from food industry [26].

A carbon fiber microelectrode covered by an ultrathin mercury film also enables sensitive and reproducible Se(IV) determination. Procedures for pre-deposition and rapid in situ mercury film formation on carbon fiber were evaluated. When mercury is deposited on carbon fibers, the number of active mercury reduction sites strongly depends on the potential used to form the film. The thickness and reproducibility of the formed film increases with the increase of negative potential. Mercury film formation was performed at potential of −1.2 V for 150 s in 5 mol L^−1^ HCl containing Hg(II) ions. The magnitude of the stripping response for Se(IV) is influenced by mercury concentration and the plating time. Enhanced diffusion flux towards such small electrode eliminates the need of stirring making analysis faster. If the stripping is realized by differential pulse scan detection limit of 1.4 × 10^−9^ mol L^−1^ can be reached in 5 mol L^−1^ HCl [27].

Bismuth film electrodes (BiFE), prepared by electroplating of a thin layer of bismuth on glassy carbon, are characterized by high sensitivity, wide cathodic potential range, good reproducibility, easy renewal of the electrode surface, insensitivity to dissolved oxygen and environmental friendliness. The behavior of selenium based on differential pulse adsorption stripping voltammetry (DPAdsSV) using a glassy carbon electrode coated with a bismuth film and *p*-aminobenzene sulfonic acid (ABSA) as a complexing agent was investigated. A well-defined stripping peak of the complex of selenium (IV) and ABSA was observed at −0.76 V (vs. SCE) in 0.15 mol L^−1^ acetate buffer at pH = 2.9 and deposition potential of −0.40 V for deposition time of 120 s. The method showed linearity in the concentration range from 2.5 × 10^−8^ to 3.8 × 10^−7^ mol L^−1^ and the detection limit during 300 s accumulation was 1.3 × 10^−9^ mol L^−1^. Trace amounts of selenium were determined in multivitamin tablets and human hair [28].

Interesting possibility for detection of trace amount of selenium represents an electropolymerization of 3,3’-diaminobenzidine on a solid gold electrode yielded a stable and water-insoluble film over a wide pH range. This polymer film was electroactive in acidic solutions. This electrode showed a very high selectivity for the accumulation of Se(IV) by complexation with aromatic o-diamine groups attached to the polymer backbone to form piaselenole. The LOD was estimated to be 9.9 × 10^−9^ mol L^−1^ with a 10 min accumulation. The relative standard deviations were 2.8% and 3.4% for selenium concentrations of 1 × 10^−6^ and 1 × 10^−7^ mol L^−1^ [29].

Trace amounts of selenium were determined by differential pulse anodic stripping voltammetry in a solution of 1 mol L^−1^ HCIO_4_ also on a gold electrode modified with methylene blue and Nafion with an accumulation potential of −240 mV and an accumulation time of 300 s. The calibration curve was linear from 1 × 10^−8^ to 1 × 10^−6^ mol L^−1^ and the calculated detection limit was 5 × 10^−9^ mol L^−1^. A Nafion layer substantially decreased interferences and lead to the detection of Se(IV) 1 000 times more sensitive compared to the bare gold electrode [30].

The microlithographic technology enabled to produce a system of ultramicroelectrodes which serves as a rapid, sensitive and microsensor for the detection of many analytes. The problem of very low current when using a separate ultramicroelectode has been solved by applying an array of ultramicroelectrodes amplifying such current response. The redox reaction of Se(IV) is kinetically faster and more reversible on gold microelectrode array than on gold macroelectrode. Relatively narrow linear dynamic concentration ranges of linearity from 1.3 × 10^−6^ to 6.3 × 10^−6^ mol L^−1^ was observed. Suggested method had the detection limit of 5.3 × 10^−9^ mol L^−1^ in 0.005 mol L^−1^ H_2_SO_4_ without the need to stir the solution due to the large diffusion mass transfer of the substance on the surface of ultramicroelectrodes [31].

The electrochemical behavior of selenium (IV) could be studied also on metallic platinum and gold disk electrode in sulfuric acid, perchloric acid and potassium chloride as supporting electrolytes. The best voltammetric behavior of the analyte was recorded on a gold electrode in perchloric acid. The presence of interferent species like copper or lead, and some biomolecules (for example bovine serum albumin) in the measured solution deformed the voltammetric response of Se(IV). Therefore SWASV was used for the quantitative detection of selenium (IV) on a 2 mm gold disk electrode, as well as on a gold interdigitated microarray electrode, on which a better linear response of selenium in the concentration range from 1 × 10^−7^ to 1 × 10^−5^ mol L^−1^ was obtained at 60 s accumulation. Due to increased diffusion transport a detection limit of 2.5 × 10^−8^ mol L^−1^ was obtained. Gold selenide alloy formation emphasizes the importance of the application of such microelectrodes in the trace detection of selenium (IV) [32].

Hrehocik et al. compared the ASV analysis of Se(IV) on three different electrode geometries—gold microdisk (diameter 10 µm), macrodisk (diameter 1.7 mm) and microarray (10 µm × 2.5 mm). All gold electrodes showed the same potential of 0.79 V stripping peak with a width of 80–90 mV. Although the flux on the microelectrodes is higher than on the macroelectrode, the detection limit of the order of 10^−8^ mol L^−1^ is similar for all three geometries. The smallest linear dynamic range was found when using a microdisk electrode. Peak current measurement accuracy is best with a macrodisk electrode [33].

For the selective detection of selenium (IV) in water a disposable electrode prepared by electrochemical synthesis of Au/ZnO nanocomposite on tin-doped indium oxide (ITO) was prepared. The nanocomposite was formed by a one-step electrochemical reduction method and shows excellent catalytic and conductive properties of gold nanoparticles as well as the high adsorption capacity of ZnO nanoparticles towards selenium. This sensor has increased selectivity and sensitivity and is resistant to interfering ions commonly found in water [34].

Another analytical approach uses a flow voltammetric detector for the rapid determination of selenium (IV) based on its reduction on a silver wire electrode. Electrochemical reduction of selenium was investigated also on a silver disk electrode, a mercury-modified glassy carbon electrode and a mercury-modified gold electrode by impedance, cyclic and differential pulse voltammetry. Se(IV) was most effectively reduced on the surface of the silver disk electrode at a potential of −0.772 V (vs. Ag/AgCl) in Britton-Robinson buffer (pH 3.5). The flow of selenium through the silver electrode was also studied. The value of the detection limit was set below 1.3 × 10^−7^ mol L^−1^. The proposed flow system does not require deposition time and it is not necessary to remove the organic substances before determining selenium in river water [35].

Hua et al. have developed a micro-electrochemical flow cell that uses carbon or gold fiber electrodes for voltammetric and amperometric analysis. The cell was tested by anodic stripping voltammetric determination of selenium (IV) on a gold fiber electrode. The formation of gold oxides on the surface of the gold electrode often results in passivation, which is eliminated by applying an anodic potential of +2 V for 10 s to this electrode in a solution containing 6 mol L^−1^ nitric acid and 2 mol L^−1^ sulfuric acid passing through the cell. The proposed cell is compatible with many electroanalytical systems due to its small size, simple structure and uncomplicated preparation [36].

An electrochemical sensor based on the SWASV method was fabricated for the simultaneous determination of As(III) and Se(IV) in water. A glassy carbon electrode was modified with gold nanoparticles (AuNPs) by electrodeposition based on potential cycling in the range from −400 mV to +1100 mV. AuNPs improve the stripping current as well as the resolution between arsenic and selenium peaks. Detection limits of 2.0 × 10^−9^ mol L^−1^ for As(III) and 2.8 × 10^−9^ mol L^−1^ for Se(IV) were achieved, with good reproducibility, in 0.1 mol L^−1^ H_2_SO_4_. The influence of Mg^2+^, Ca^2+^, Na^+^, K^+^, Cu^2+^, Cd^2+^ ions was found to be statistically negligible, even when present in a large excess. Proposed method was applied to real water samples and validated by inductively coupled plasma optical emission spectroscopy [37].

Arsenic (III) together with selenium (IV) was determined in environmental matrices by differential pulse cathodic stripping voltammetry as well as copper (II), lead (II), cadmium (II), zinc (II) and manganese (II) by differential pulse anodic stripping voltammetry using a stationary mercury electrode in ammonia and ammonium chloride buffer, pH 9.0. The method was verified by analysis of some standard reference materials and seawater real samples. In seawater, strong interference was shown at a selenium to arsenic molar concentration ratio of 220.5 in the case of simultaneous DPCSV detection of As(III) and Se(IV). The value of the detection limit for each element was about 10^−9^ mol L^−1^ with a relative standard deviation of less than 5% [38].

There is currently a growing need to monitor heavy metals in complex matrices that are involved in the food chain. Locatelli et al. in their work described the sequential detection of Cu(II), Pb(II), Cd(II) and Zn(II) by square-wave anodic stripping voltammetry, As(III) and Se(IV) using square-wave cathodic stripping voltammetry as well as Mn(II) and Fe(III) using square-wave voltammetry in whole grain, wheat and corn flour on HMDE in 0.5 mol L^−1^ HCl as well as in a buffer solution of ammonia and ammonium chloride at pH 8.8 as supporting electrolytes. Lower detection limits (10^−9^–10^−10^ mol L^−1^) were obtained when the area of stripping peak was used for calibration instead the peak current. When the signals interfere with each other, the standard addition method minimizes any matrix effects and greatly improves the resolution of the voltammetry signals. The relative standard deviations in all cases were less than 6%. The accuracy of the results was confirmed by the atomic absorption spectrometry (AAS) technique [39].

**Figure 2:**
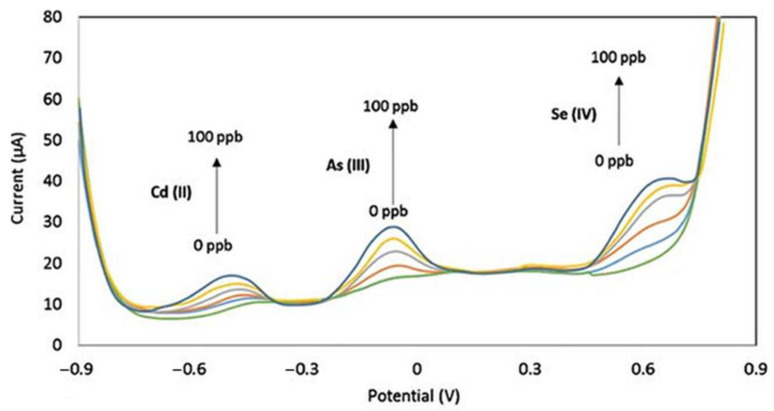
SWASV response of AuNS-modified SPCE electrode for simultaneous detection of Cd(II),As(III)and Se(IV) in concentration range from 0 to 100 ppb (around 10^−6^ mol L^−1^) [40].

Gold nanostar (AuNS) modified carbon screen-printed electrodes (SPCE) in Britton-Robinson buffer at pH = 2 were also applied for the simultaneous determination of trace Cd(II), As(III) and Se(IV) by SWASV. The above mentioned electrolyte provides a wide potential window that allows good separation of all three peaks, thus enhancing the selectivity of the determination (Figure 2). Electrochemical impedance and spectral tests confirmed a significant reduction in the charge transfer resistance of AuNS/SPCE (0.8 kΩ) compared to bare SPCE (2.4 kΩ). By modifying the electrode, the increased surface area and low charge transfer resistance resulted in the high sensitivity of the assay. The signal of the analyses increased linearly with increasing concentration till 1.3 × 10^−6^ mol L^−1^, while the response of cadmium was not affected by the presence of arsenic and selenium. LOD values of 1.4 × 10^−8^ mol L^−1^ for Cd(II), 1.1 × 10^−8^ mol L^−1^ for As(III) and 2.0 × 10^−8^ mol L^−1^ for Se(IV) were obtained. Approximately 40% reductions in peak height in the simultaneous detection of As(III) and Se(IV) were due to the formation of electrochemically inactive arsenic selenide As_2_Se_3_ in the deposition step. The proposed method is reliable for surface water [40]. A survey of electrochemical methods for the determination of selenium with their analytical performance is summarized in Table 1.

## 4. Conclusions

The presented review paper deals with the electrochemical detection of selenium in environmental and biological matrices using various electrode materials. Cathodic, anodic and adsorption stripping voltammetry in differential pulse or square-wave mode are mainly used to determine the various forms of selenium. The electrochemical behavior of inorganic Se in acidic electrolytes was studied on a hanging mercury drop electrode, a film mercury electrode, glassy carbon, carbon paste, pencil graphite, screen-printed carbon electrodes and carbon microelectrodes. Solid electrodes based on silver, platinum and gold have also been applied for its detection, including silver, platinum and gold disk electrodes, gold ultramicroelectrodes, microelectrodes and microelectrode arrays. The surface of solid working electrodes has been modified with various classes of modifiers such as mercury films, bismuth films, polymeric and organic modifiers and nanomaterials. These modifiers make it possible to achieve better sensor analytical characteristics, i.e., lower detection limits, higher sensitivity and wider dynamic linear measurement ranges. The most sensitive voltammetric procedure for the determination of selenium is cathodic stripping voltammetry in the presence of Rh(III) ions on HMDE, where the lowest limit of detection for selenium 6 × 10^−12^ mol L^−1^ was reached. Fabrication of microelectrodes and their arrays increase functionality of bare boron- doped diamond (BDD) materials and open the door to targeted sensing without the necessity of BDD surface modification. Further miniaturization of BDD-based devices for functions required in in-vitro/in-vivo analysis or detection in micro-scale analytical systems can be expected. 

## Figures and Tables

**Table 1:** Comparison of analytical performance of electrochemical sensors for the detection of selenium.

Method ^1^/ Analyte	Electrode ^2^	LOD ^3^ (mol L^−^^1^)	LDR ^4^ (mol L^−^^1^)	Sensitivity	Ref.
CSV/Se(IV)	HMDE	4 × 10^−10^	1 × 10^−9^–4 × 10^−8^	9.65 nA nL mol^−1^	[12]
CVS/Se(IV)	HMDE	7.8 × 10^−10^	2 × 10^−9^–2 × 10^−7^	0.74 nA nL mol^−1^	[13]
SWCSV /Se(IV)	HMDE	8 × 10^−12^	1 × 10^−11^–1 × 10^−6^	NR ^5^	[14]
CSV/Se(II)	HMDE	6 × 10^−12^	NR	NR	[15]
SWCSV/Se(IV)	HMDE	1.3 × 10^−11^	6.3 × 10^−10^–6.3 × 10^−7^	NR	[16]
CSV/Se(IV), Se(VI)	HMDE	3.8 × 10^−10^ NR	1.3 × 10^−8^–1.3 × 10^−6^ NR	NR NR	[17]
CSVwith AFS/Se(IV), Se(VI)	HMDE	4.4 × 10^−9^ NR	1.3 × 10^−8^–1.9 × 10^−6^ NR	NR NR	[18]
SWCSV /Se-U, Se-CM	HMDE	2.5 × 10^−8^ 3.8 × 10^−9^	NR	78.96 nA µL mol^−1^ 394.8 nA µL mol^−1^	[19]
DPCSV /Se(IV)	TFME/GCE	9.5 × 10^−10^	6.3 × 10^−8^–3.8 × 10^−7^	NR	[20]
SWCSV /Se(IV)	Cu/TFME/GCE	8 × 10^−10^	1 × 10^−9^–1 × 10^−6^	NR	[21]
SWCSV /Se(IV)	ODAB/Tosflex /TFME/GCE	1.3 × 10^−9^	6.3 × 10^−9^–6.3 × 10^−7^	118.44 µA µL mol^−1^	[22]
SWASV /Se(IV)	GCE/AuNDs /P-rGO	9 × 10^−10^	3 × 10^−9^–3 × 10^−7^	45.38 µA µL mol^−1^	[23]
DPAdsCSV /Se(IV)	Bi/TFME/GCE	8.9 × 10^−10^	2.5 × 10^−8^–6.3 × 10^−7^	24.69 µA µL mol^−1^	[24]
AdsCSV /Se(IV)	CPE	1.25 × 10^−7^	3 × 10^−7^–2 × 10^−5^	0.58 A L mol^−1^	[25]
SWV/Se(IV)	PPy-ADH /CuNPs/PGE	1.7 × 10^−8^	5 × 10^−8^–1.1 × 10^−7^	0.5464 µA nL mol^−1^	[26]
DPCSV /Se(IV)	MF/CFME	1.4 × 10^−9^	2 × 10^−9^–3.8 × 10^−8^	NR	[27]
DPAdsSV /Se(IV)	BiFE/GCE	1.3 × 10^−9^	2.5 × 10^−8^–3.8 × 10^−7^	15.62 µA µL mol^−1^	[28]
DPASV /Se(IV)	PolyDAB /AuE	9.9 × 10^−9^	2 × 10^−8^–1.3 × 10^−6^	4.079 µA µL mol^−1^	[29]
DPASV /Se(IV)	MB-N/AuE	5 × 10^−9^	1 × 10^−8^–1 × 10^−6^	11.5 µA µL mol^−1^	[30]
SWASV /Se(IV)	AuUMEAs	5.3 × 10^−9^	1.3 × 10^−6^–6.3 × 10^−6^	NR	[31]
SWASV /Se(IV)	AuIDMEA	2.5 × 10^−8^	1 × 10^−7^–1 × 10^−5^	100 µA µL mol^−1^ cm^−2^	[32]
ASV /Se(IV)	AuMaDE	2 × 10^−8^	1 × 10^−7^–1 × 10^−5^	2.2 A L mol^−1^	[33]
SWASV /Se(IV)	Au/ZnO/ITO	3.7 × 10^−8^	6.3 × 10^−8^–1.3 × 10^−6^	15 µA µL mol^−1^	[34]
Flow cell/Se(IV)	AgWE	1.3 × 10^−7^	2.5 × 10^−6^–2 × 10^−5^	NR	[35]
ME flow cell/Se(IV)	AuFE	NR	NR	NR	[36]
SWASV /Se(IV)	AuNPs/GCE	2.8 × 10^−9^	1.3 × 10^−7^–1.5 × 10^−4^	1.46 A L mol^−1^	[37]
DPCSV /Se(IV)	SMDE	2.1 × 10^−10^	NR	48.3 A L mol^−1^	[38]
SWCSV /Se(IV)	HMDE	6.5 × 10^−10^	NR	NR	[39]
SWASV /Se(IV)	AuNS/SPCE	2.0 × 10^−8^	5.3 × 10^−8^–1.3 × 10^−6^	10 µA µL mol^−1^	[40]

^1^ CSV: cathodic stripping voltammetry, SWCSV: square-wave cathodic stripping voltammetry, CSV with AFS: cathodic stripping voltammetry with automated flow system, Se-U: selenourea, Se-CM: selenocystamine, DPCSV: differential pulse cathodic stripping voltammetry, SWASV: square-wave anodic stripping voltammetry, DPAdsCSV: differential pulse adsorption cathodic stripping voltammetry, AdsCSV: adsorption cathodic stripping voltammetry, SWV: square-wave voltammetry, DPAdsSV: differential pulse adsorption stripping voltammetry, DPASV: differential pulse anodic stripping voltammetry, ASV: anodic stripping voltammetry, ME: microelectrochemical, ^2^ HMDE: hanging mercury drop electrode, TFME: thin film mercury electrode, GCE: glassy carbon electrode, ODAB: photooxidized 3,3’-diaminobenzidine, Tosflex: perfluorinated polymeric anion exchanger, AuNDs: gold nanodendrites, P-rGO: perforated-reduced graphene oxide, CPE: carbon paste electrode, PPy-ADH: polypyrrole-acetophenone (2,4-dinitrophenyl)hydrazone, CuNPs: copper nanoparticles, PGE: pencil graphite electrode, MF: mercury film, CFME: carbon fiber microelectrode, BiFE: bismuth film electrode, PolyDAB: poly(3,3’-diaminobenzidine), AuE: gold electrode, MB-N: methylene blue-Nafion, AuUMEAs: gold ultramicroelectrode arrays, AuIDMEA: gold interdigitated microelectrode array, AuMaDE: gold macrodisk electrode, Au/ZnO: nanocomposite of gold and zinc oxide, ITO: tin-doped indium oxide, AgWE: silver wire electrode, AuFE: gold fiber electrode, AuNPs: nanoparticles of gold, SMDE: stationary mercury drop electrode, AuNS: gold nanostar, SPCE: screen-printed carbon electrode, ^3^ LOD: limit of detection, ^4^ LDR: linear dynamic range, ^5^ NR: not reported.

## Data Availability

The data presented in this study are available on request from the corresponding author.
